# Bioengineered Hybrid Rep 2/6 Gene Improves Encapsulation of a Single-Stranded Expression Cassette into AAV6 Vectors

**DOI:** 10.3390/genes14101866

**Published:** 2023-09-26

**Authors:** Marcos Tejero, Ozgun F. Duzenli, Colin Caine, Hisae Kuoch, George Aslanidi

**Affiliations:** 1 Hormel Institute, University of Minnesota, 801 16th Avenue NE, Austin, MN 55455, USA; marcostejero86@gmail.com (M.T.);; 2 Masonic Cancer Center, University of Minnesota, Minneapolis, MN 55455, USA; 3 Institute Molecular Virology, University of Minnesota, Minneapolis, MN 55455, USA

**Keywords:** adeno-associated virus, Rep gene, genome encapsulation, upstream production

## Abstract

The production of clinical-grade recombinant adeno-associated viral (AAV) vectors for gene therapy trials remains a major hurdle in the further advancement of the gene therapy field. During the past decades, AAV research has been predominantly focused on the development of new capsid modifications, vector-associated immunogenicity, and the scale-up vector production. However, limited studies have examined the possibility to manipulate non-structural components of AAV such as the Rep genes. Historically, naturally isolated, or recombinant library-derived AAV capsids have been produced using the AAV serotype 2 Rep gene to package ITR2-flanked vector genomes. In the current study, we mutated four variable amino acids in the conservative part of the binding domain in AAV serotype 6 Rep to generate a Rep2/6 hybrid gene. This newly generated Rep2/6 hybrid had improved packaging ability over wild-type Rep6. AAV vectors produced with Rep2/6 exhibited similar in vivo activity as standard AAV6 vectors. Furthermore, we show that this Rep2/6 hybrid also improves full/empty capsid ratios, suggesting that Rep bioengineering can be used to improve the ratio of fully encapsulated AAV vectors during upstream manufacturing processes.

## 1. Introduction

Recombinant adeno-associated viruses (AAVs) are one of the most widely used vector systems for gene therapy applications, and the number of clinical trials using gene therapies are increasing. To date, several AAV-based therapies have been approved for commercialization [1]. Glybera, an AAV1 vector for lipoprotein lipase deficiency; Luxturna, an AAV2 vectors for Leber’s congenital amaurosis [2]; Zolgensma and Elevidys, an AAV9 vectors for spinal muscular atrophy type 1 [3] and Duchenne muscular dystrophy [4], correspondingly; Hemgenix and Roctavian [5], an AAV5 vectors for Hemophilia B [6] and Hemophilia A, respectively.

Although AAV production methods are well established [7], optimizing processes and scale-up operations are becoming an important focus to obtain high yields, superior quality, and purity of the vectors suitable for clinical trials [8,9]. Regardless of AAV production methods, around 50% of generated viral particles fail to package AAV genomes. These “empty capsids” are considered one of the main byproducts during AAV production [10], and, although the effects of these empty capsids are not fully understood, their presence increases the total vector load necessary to obtain beneficial clinical effects [11]. Thus, reducing the number of empty capsids can help enhance efficiency of AAV therapies, and lower the risk of inactivation of the therapy by preventing the potential elucidation of capsid related immune responses. The basis of the current AAV manufacturing relies on the separation of empty capsids in the downstream purification process [12,13,14,15]. However, a higher yield of the functional AAV vectors could be possible with a more efficient upstream process.

The wild-type AAV genome is a single-stranded DNA (approximately 4700 nucleotides) flanked by 145 base-pair-inverted terminal repeats (ITR) that form base-paired harpings [16]. The genome is organized into two open reading frames (Rep and Cap), -structural and structural proteins, respectively [17].

The Rep gene encodes four Rep proteins responsible for the viral cycle. Rep78 and Rep68 are generated by one transcript from the p5 promoter and are involved in viral genome excision, rescue, replication, and integration, as well as the regulation of gene expression [18,19]. Rep52 and Rep40 are generated by another transcript from the p19 promoter and are essential for genome packaging into pre-formed viral capsids [20].

All AAV Rep proteins (Rep78, 68, 52 and 40) share the central domain with a nuclear localization signal [21] and have ATP-dependent helicase activity [22,23]. Rep52/40 helicase activity may translocate single-stranded DNA genomes through the five-fold symmetry axis of pre-formed AAV capsids [20,24,25]. Besides the helicase domain, Rep78 and Rep68 have a DNA binding domain with endonuclease activity located in the N-terminal region [23] that binds to the Rep binding element located in the ITRs to initiate genome replication [26,27,28] after DNA cleavage on the terminal resolution site (*trs*) [29]. Rep proteins have been described to be members of the HUH endonucleases superfamily that include a “His-Hydrophobic-His” motif and one or two Tyr residues (Y1 or Y2 subfamilies) in the catalytic domain required for ssDNA breakage [30].

The Cap gene encodes three structural viral proteins (VP1, VP2, and VP3) driven by the p40 promoter in approximately a 1:1:10 ratio [31]. It also encodes two short off-frame accessory proteins: the assembly activating protein (AAP) [32], which works as a scaffold protein during capsid assembly [33]; and the recently discovered membrane-associated accessory protein (MAAP) [34].

AAV vectors carry the gene of interest flanked by ITRs, and the Rep and Cap genes are supplied in *trans* for vector production [35]. Genome replication and capsid formation are two parallel events that conclude with genome encapsulation to package mature AAV vectors [36]. Nevertheless, the many empty capsids formed during production could also be packaged with better upstream processes. Historically, AAV vectors were produced with the AAV serotype 2 Rep gene and ITRs with the corresponding CapX gene to generate different serotypes. AAV serotype 2 Rep (Rep2) can package expression cassettes flanked with ITR2 into all serotype capsids, but the encapsulation efficiency is relatively low, which results in more empty capsids [37]. Genome packaging is considered a limiting step for AAV vector production and requires improvement to ensure superior quality of AAV vectors for clinical application. However, few studies have addressed the use of alternative or engineered Rep [38,39].

The current study was designed to evaluate the ability of different Rep proteins to encapsulate the expression cassette flanked with ITR2 or ITR6 into AAV6 vectors. We compared AAV productivity using AAV serotype 6Rep (Rep6), AAV serotype 2 Rep (Rep2) and an engineered Rep2/6 hybrid. The Rep N-terminal region contains a DNA binding site that interacts with the ITR-specific Rep Recognition Sequence (RRS) [40,41]. We anticipated that endogenous p5, p19 and p40 promoters in Rep6 and ITR6-specific RRS should be advantageous for AAV6 production. We showed, however, that Rep6 activity alone was insufficient in packaging the expression cassette into AAV6 vectors, in part due to low Rep78/68 expression. Site-directed mutagenesis of the four critical amino acids, V65T, Q66E, L90M, and I92V to the corresponding Rep2 residues significantly improved Rep6 activity, and thus, the ratio of full vs. empty capsids in purified AAV6 vectors. We also confirmed activity of the AAV6 vectors generated with the mutated Rep gene by in vivo luciferase assay after the intramuscular injection of these vectors. Thus, our study confirmed that optimization of the Rep gene could increase production of fully encapsulated AAV particles.

## 2. Materials and Methods

### 2.1. Cell Culture

Human embryonic kidney cells (HEK293) were maintained in complete Dulbecco’s Modified Eagle Medium (Thermo Fisher/Gibco, Manassas, VA, USA) supplemented with 10% fetal bovine serum (Thermo Fisher/Gibco Manassas, VA, USA), 1% penicillin and streptomycin (Genesee Scientific, El Cajon, CA, USA) at 37 °C and 5% CO_2_.

### 2.2. Animals

Six- to ten-week-old male C57BL6 mice were obtained from Jackson Laboratories (Bar Harbor, ME, USA). All experiments with mice were performed according to the principles of the National Research Council’s Guide for the Care and Use of Laboratory Animals, with approval from the University of Minnesota Institutional Animal Care and Use Committee (IACUC).

### 2.3. Plasmids, Cloning, and Rep Mutant Generation

A Rep6Cap6 plasmid was generated by replacing the Rep2 (Sequence ID: UXD78434.1) gene from a Rep2Cap6 plasmid [42] with a PCR-amplified Rep6 gene using the wild-type AAV6 genome as a template (Sequence ID: AF028704.1). Rep6 mutant genes were generated by site-directed mutagenesis [42,43] using the Rep6/Cap6 plasmid as a template with the following primers: Rep6 V65T (Forward primer 5′-CTGCAGCGCGACTTCCTGACCCAGTGGCGCCGCGTGAGT-3′ and Reverse primer 5′-ACTCACGCGGCGCCACTGGGTCAGGAAGTCGCGCTGCAG-3′) to change Valine (GTC) to Threonine (ACC); Rep6 Q66E (Forward primer 5′-CAGCGCGACTTCCTGGTCGAGTGGCGCCGCGTGAGTAAG-3′ and Reverse primer 5′-CTTACTCACGCGGCGCCACTCGACCAGGAAGTCGCGCTG-3′) to change Glutamine (CAG) to Glutamic acid (GAG); Rep6 L90M (Forward primer 5′-GGCGAGTCCTACTTCCACATGCATATTCTGGTGGAGACC-3′ and Reverse primer 5′- GGTCTCCACCAGAATATGCATGTGGAAGTAGGACTCGCC-3′) to change Leucine (CTC) to Methionine (ATG); Rep6 I92V (Forward primer 5′-TCCTACTTCCACCTCCATGTTCTGGTGGAGACCACGGGG-3′ and Reverse primer 5′-CCCCGTGGTCTCCACCAGAACATGGAGGTGGAAGTAGGA-3′) to change Isoleucine (ATT) to Valine (GTT). Red color indicates changed nucleotides in Rep6 sequence.

Briefly, primers containing single nucleotide mutations were used to introduce point mutations in the AAV6 Rep gene. Two-step PCR was performed as described previously with high-performance Velocity DNA polymerase (Bioline, London, UK) [42]. The PCR product was purified with a DNA Clean and Concentrator Kit (Zymo Research, Irvine, CA, USA) and digested with DpnI restriction enzyme (New England BioLabs, Ipswich, MA, USA) prior to transformation. Mutagenesis was confirmed with sequencing prior to AAV packaging.

### 2.4. AAV Production and Purification

All AAV vectors were packaged with a single-strain expression cassette harboring chicken β-actin promoter-driven fusion of firefly luciferase, and yellow fluorescent protein (YFP) [44] or green fluorescent protein (GFP). Vectors were produced by the triple transfection method as previously described [45,46]. Briefly, HEK293 cells were co-transfected with three plasmids by polyethylenimine (Polyscince, Warrington, PA, USA) and were harvested 72 h post-transfection. Cell lysates were subjected to three rounds of freeze–thaw and then digested with 50 U/ml benzonase (Sigma, St. Louis, MO, USA) at 37 °C for 1 h. Viral vectors were purified by iodixanol (Sigma, St. Louis, MO, USA) gradient ultracentrifugation followed by ion-exchange chromatography using HiTrap Q HP (Cytiva, Piscataway, NJ, USA) with TRIS-NaCl buffer and concentrated by centrifugation using centrifugal spin concentrators with 150 K molecular-weight cutoff (Orbital Biosciences, Topsfield, MA, USA). Viral vectors were finally resuspended in 500 μL PBS.

### 2.5. Quantitative PCR Analysis for AAV Titration

AAV titers were quantified by relative to standard qPCR analysis using chicken b-actin promoter-specific primers (Forward primer 5′-TCCCATAGTAACGCCAATAGG-3′ and reverse primer 5′-CTTGGCATATGATACACTTGATG-3′) with a SensiFast SYBR No-ROX Kit (Bioline, London, UK). Aliquots from AAV productions were digested with TURBO DNase (Thermo Fisher Scientific, Waltham, MA, USA) to remove DNA contaminants followed by proteinase K (Invitrogen, Waltham, MA, USA) digestion to release AAV genomes from AAV capsids.

### 2.6. Western Blots

Rep and capsid viral protein (VP) expression was analyzed by Western blotting on production cell lysates or purified vectors [47]. Briefly, cell lysates from production cells were separated on 10% polyacrylamide/SDS gels and transferred to nitrocellulose membranes. Primary antibodies anti-VP Anti-AAV VP1+VP2+VP3; clone B1 (mouse mAb 1:2000) or Anti-AAV Replicase (Rep 78, 68, 52, 40); clone 259.5 (mouse mAb 1:1000) (ARP, Waltham, MA, USA), followed by secondary horseradish peroxidase-linked antibodies (1:1000) (Cell Signaling Technology, Danvers, MA, USA) were used to visualize protein expression. Precision Plus Kaleidoscope Prestained Protein Standards (BioRad, Hercules, CA, USA) was used to relate molecular weight of viral proteins.

### 2.7. In Vivo Luciferase Activity

C57BL6/J mice were intramuscularly injected with 1 × 10^10^ vg/mice with AAV vectors encoding the luciferase-YFP fusion gene. Expression was monitored weekly with IVIS Lumina S5 Imaging System (Perkin Elmer, Waltham, MA, USA) [43,44]. Bioluminescence images were obtained by intraperitoneal injection (250 µL/mice) of D-luciferin substrate (Promega, Madison, WI, USA) and analyzed using Living image v4.7.4 software (Perkin Elmer, Waltham, MA, USA) according to manufacture protocol. Background was normalized by imaging of non-injected animals.

### 2.8. Transmission Electron Microscopy (TEM)

An AAV sample (3 µL) was placed on a 150 mesh copper grid coated with 5–6 nm Formvar/carbon EMS CF150-Cu film (Electron Microscopy Sciences, Hatfield, PA, USA) for 1 min. The grid was washed with 3 drops of 6 μL distilled water. Excess water was removed with Whatman filter paper. The sample was negatively stained with 6 μL 0.75% uranyl acetate (SPI Supplies, West Chester, PA, USA) for 30 s. Excess staining solution was removed with Whatman filter paper and the grid was dried at room temperature. We captured 6–12 electron micrographs of AAV capsids using a Tecnai G2 Spirit BioTwin electron microscope (FEI, Hillsboro, OR, USA) equipped with a 4K CCD Gatan Ultrascan camera (Gatan, Pleasanton, CA, USA) at an accelerating voltage of 120 kV and a nominal magnification of 30,000× and 49,000×.

### 2.9. Statistical Analyses

All data are shown as mean ± SEM. Statistical significance was determined by unpaired two-tailed *t*-tests or ANOVA using GraphPad Prism software 7.3 (GraphPad Software, La Jolla, CA, USA). We set statistical significance as a *p* value < 0.05.

## 3. Results

### 3.1. Rep2/6 Hybrids Improve Wild-Type Rep6 Activity

The AAV3 Rep gene (Rep3) can package AAV vectors flanked by ITR2 or ITR3 [39]. However, similar results were not obtained with Rep6, which failed to package AAV6 vectors either with ITR2 or ITR6.

**Figure 1:**
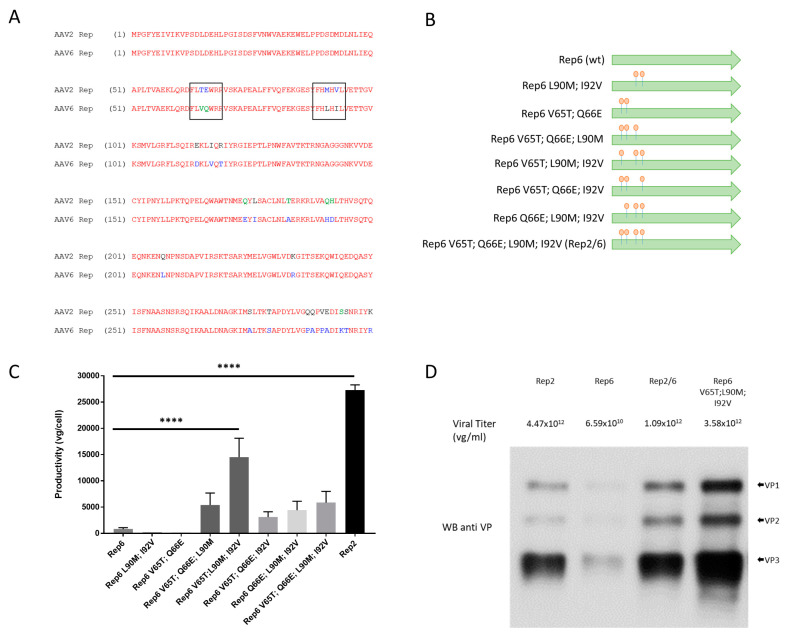
Rep6 mutagenesis to generate Rep2/6 hybrids: (**A**) Rep6 and Rep2 protein sequences alignment of DNA binding region). Black squares located in positions 65/66 and 90/92 indicate the difference in sequences between Rep6 and Rep2. (**B**) Diagram of Rep6 to Rep2 amino acids substitutions. Green color corresponds with Rep6 sequences while orange color corresponds with Rep2 amino acids. (**C**) qPCR titration with promoter specific primers of AAV6-luciferase vectors produced with different Rep6 mutants from small-scale (6-well plate) production presented as viral genome (vg) copies per cell. (**D**) Western blots of anti VP and viral titer (qPCR) from purified AAV6-luciferase vectors produced in 25 × 15 cm plates.

The binding domain of AAV6 Rep78 protein differs from the AAV2 Rep78 protein (Figure 1A). Here, we mutated four distinct residues in the conservative region about ninety amino acids downstream from the start codon and generated two double (V65T/Q66E and L90M/I92V); four triple (V65T/Q66E/L90M, V65T/L90M/I92V, V65T/Q66E/I92V, and Q66E/L90M/I92V); and one quadruple (V65T/Q66E/L90M/I92V) Rep6 mutants maintaining wild-type Rep52/40 and Rep6 p19 and p40 promoter boosted the integrity. To evaluate the effects of each amino acid in Rep6 packaging activity, we produced AAV6 vectors carrying the ITR2-luciferase-GFP reporter gene to compare productivity for all mutant combinations (Figure 1B).

Rep6 double mutants did not restore AAV6 production (Figure 1C), while the triple and quadruple mutants increased AAV6 yield significantly compared with wild-type Rep6. The Rep6-V65T/L90M/I92V triple mutant produced a vector yield per cell two times lower than that of Rep2. Our results suggest a failure of the Rep6 binding regions activity during AAV production.

Next, we selected four different Rep constructs to further evaluate AAV production and encapsulation. Purified vectors were analyzed either by qPCR or Western blots to evaluate productivity, presence, and ratio of viral protein subunits (Figure 1D). As expected from our preliminary results, Rep6 produced a yield approximately 100 times lower than Rep2, but the triple mutant Rep6-V65T/L90M/I92V and the quadruple mutant Rep6-V65T/Q66E/L90M/I92V (called hybrid Rep2/6 hereafter) produced titers within a similar range as Rep2. We observed no differences in AAV purification profiles in terms of binding/elution to ion-exchange columns. To test capsid integrity and VP protein proportion, we loaded equal aliquots of different purified vectors into SDS-PAGE columns. All four AAV6 vectors showed the same capsid protein distribution on Western blots (Figure 1D), although the total amount of detected VP proteins normalized to vector genome yields differed. This discrepancy might be partially explained by different full/empty capsid ratios.

**Figure 2:**
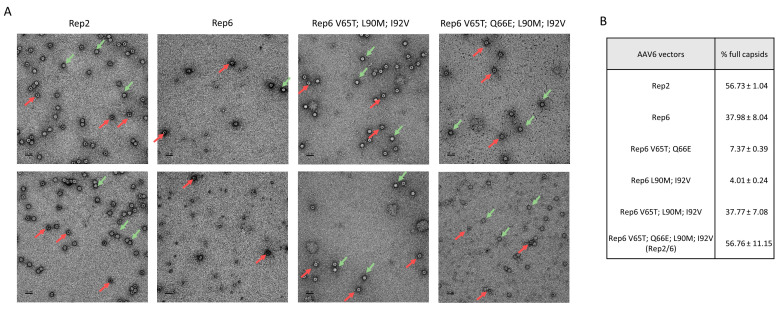
Characterization of AAV6 vectors produced with different Rep genes: (**A**) Representative TEM micro images for each variant of Rep used to package AAV6-luciferase vectors. Green arrows indicate fully encapsulated vectors (open circle) and red arrows indicate empty vectors (circle with black dot). (**B**) Percentage of full vectors counted from 2 independent preparations with the corresponding Rep gene. Representative images are shown.

We examined packaging efficiency of different Rep proteins by electron microscopy to compare the ability to encapsulate AAV genomes into pre-formed capsids. Rep6 produced a limited amount of fully encapsulated AAV6 vectors compared with Rep2, and this ratio was not improved by any double mutations on Rep6 (Figure 2). Furthermore, the yield of AAV6 vectors produced with the Rep6 double mutants were approximately 100 times lower than the Rep2-produced vectors (Figure 1C). Nevertheless, triple mutant Rep6-V65T/L90M/I92V partially restored Rep6 packaging capability (37% of full capsid) and produced a genomic titer similar to Rep2-produced vectors. Surprisingly, the Rep2/6 hybrid (or quadruple Rep6 mutant), which showed lower yields, restored packaging ability and produced up to 50% of fully encapsulated AAV vectors similar to Rep2 (Figure 2). Our data suggest the importance of the Rep N-terminus region, which is involved in Rep78/68 expression and DNA binding.

### 3.2. AAV6 Vector Activity Is Independent of the Rep Production System

**Figure 3:**
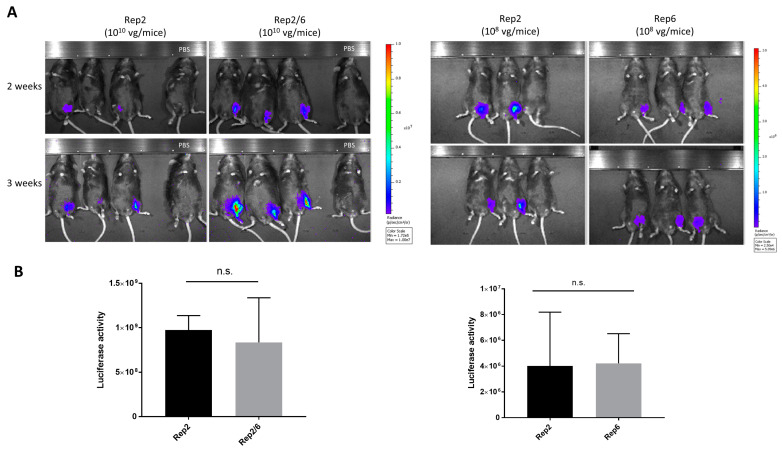
In vivo activity of AAV vectors produced with different Rep genes: Intramuscular administration of AAV6-luciferase (10^10^ vg/animal) produced with Rep2 or Rep2/6 and (10^8^ vg/animal) produced with Rep2 or Rep6 in C57BL6 mice (*n* = 3). (**A**) In vivo imaging of luciferase activity 2 and 3 weeks after administration. (**B**) Luciferase activity quantification at the endpoint of experiment. Representative images are shown. The color bar and min/max value represents the range of luminescence intensity (radiance p/sec/cm^2^sr) automatically calculated by Living image v4.7.4 software. n.s.—non significant.

To test transduction efficiency, C57BL6 mice were intramuscularly injected (*n* = 3/group) with Rep2, Rep2/6, and Rep6 produced AAV6 vectors carrying ITR2-luciferase-GFP as a reporter gene. Due to the low yield of Rep6 produced vectors, we used two different matching doses for the Rep2, Rep2/6 (1 × 10^10^ vg/animal) and the Rep2, Rep6 (1 × 10^8^ vg/animal) groups. Two experiments were performed using independent viral productions, and whole-body in vivo luciferase activity was followed for 3 weeks after vector injection (Figure 3A). We observed no significant differences in luciferase activity between both vectors (Rep2/6 and Rep6) compared to Rep2 control mice (Figure 3B). This result suggests that transduction ability of AAV6 vectors is autonomous of the Rep gene used for production.

### 3.3. Rep2/6 Hybrid Restores Rep78/68 Expression Diminished in Rep6

**Figure 4:**
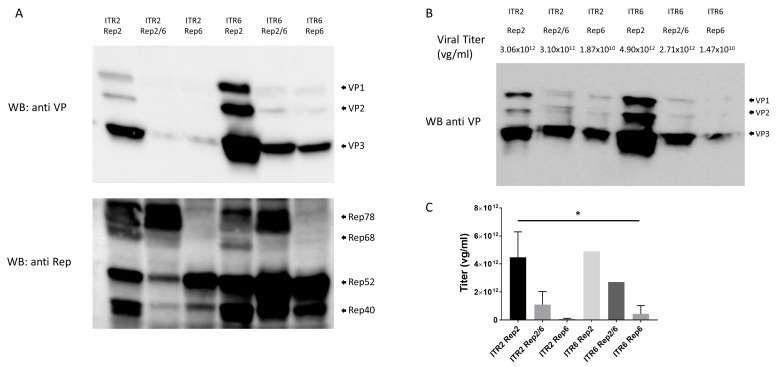
Characterization of AAV6 vectors produced with different Rep genes and ITRs: (**A**) Western blot with anti-VP and anti-Rep antibodies on equal volume of HEK293 cells production lysates. (**B**) Viral titration (qPCR) and VP ratio (Western blot with anti-VP antibodies) from equal volume of purified vectors. (**C**) Viral titration for different preparations of AAV6 vectors by qPCR from 3 independent viral productions and presented in viral genome (vg) copies per mL.

According to our previous data, Rep6 was not able to package AAV6 vectors, but all were produced using commonly used ITR2 flanked genomes. To determine if this effect stemmed from ITRs, we packaged separate ITR2 and ITR6 flanked genomes in AAV6 vectors, produced with Rep2, Rep6, and Rep2/6 hybrids. First, we analyzed cellular lysates for VP and Rep protein expression and distribution by Western blots in packaging HEK293 cells at their harvest point (Figure 4A). Rep6 produced lower amounts of Rep78/68 proteins compared with Rep2, but that expression level was restored with Rep2/6 mutations in the N-terminus region. These results suggest that Rep6 did not efficiently package AAV6 vectors due to a lack of Rep78/68 expression. On the other hand, all vectors packaged with ITR6 showed higher amounts of VP protein compared with the homologous Rep packaged with ITR2, suggesting that encapsulation of the viral genome is lower with ITR6 vectors than with ITR2-contained vectors (Figure 4A). Similar to observations in production cell lysates, purified AAV6 vectors produced with Rep2 and ITR6 yielded higher amounts of protein compared with other vectors, leading to a discrepancy between total VP proteins and viral titers (Figure 4B). Moreover, all ITR6- and ITR2 contained vectors showed the same VP ratios. At the same time, the purified AAV vectors, packaged with ITR2 and ITR6 with the same Rep gene, showed similar viral titers (Figure 4C).

### 3.4. AAV6 Vector Activity Is ITR-Independent

**Figure 5:**
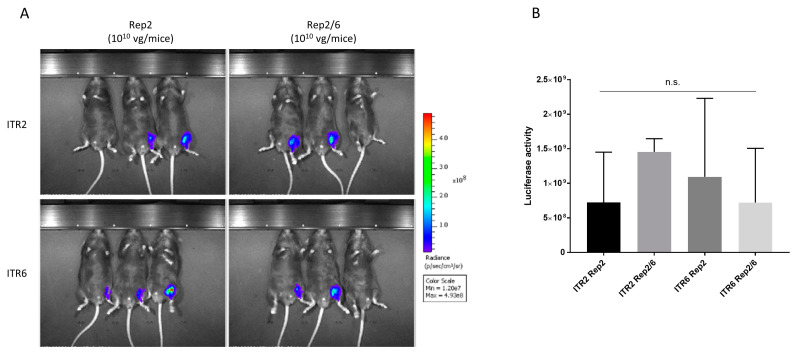
In vivo activity of AAV vectors produced with different Rep genes and ITRs: Intramuscular administration of ITR2/ITR6 AAV6-luciferase (10^10^ vg/animal) produced by Rep2 or Rep2/6 in C57BL6 mice (*n* = 3). (**A**) In vivo imaging of luciferase activity 3 weeks after administration. (**B**) Luciferase activity quantification at the endpoint of experiment. Representative images are shown. The color bar and min/max value represents the range of luminescence intensity (radiance p/sec/cm^2^sr) automatically calculated by Living image v4.7.4 software. n.s.—non significant.

The transduction efficiency of AAV6 vectors was evaluated in vivo in ITR2 and ITR6-containing expression cassette vectors. ITR2 or ITR6 flanked luciferase-GFP vectors produced with Rep2 or hybrid Rep2/6 were intramuscularly injected into C57BL6 mice (1 × 10^10^ vg/animal). Whole-body imaging was performed weekly for 3 weeks to measure luciferase activity (Figure 5). All four AAV6 vectors showed similar activity, with no statistically significant differences in in vivo transduction, suggesting that transduction efficiency is independent of the production system.

### 3.5. Rep2/6 Hybrids Rescue Rep6 Encapsulation Capability of ITR2 and ITR6

**Figure 6:**
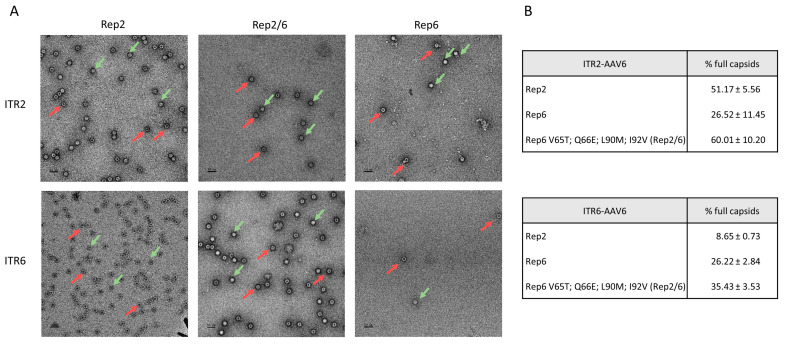
Microscopic characterization of AAV6 vectors produced with different Rep genes: (**A**) Representative electron microscopy micrographs for each variant of Rep protein used to package AAV6. Green arrows indicate fully encapsulated (open cycle) vectors and red arrows indicate empty (circle with black dot) vectors. (**B**) Percentage of full vectors counted from 3 independent preparations with the corresponding Rep gene. Representative images are shown.

Finally, we examined the ability of Rep6 and Rep2/6 vectors to package ITR6 or ITR2 flanked expression cassettes using electron microscopy. Three independent viral productions were analyzed for each group and the percentage of fully encapsulated vectors was quantified using 5 to 10 images with 30,000× magnification for each packaging condition. Representative images with higher magnification (49,000×) are shown in Figure 6. For both ITR2 and ITR6, Rep6-produced vectors showed low vector genome yield (approximately 26% of all capsids were full). ITR6 packaged with Rep2 showed an exceptionally low amount of fully encapsulated vectors (below 10%). However, hybrid Rep2/6 restored Rep6 encapsulation abilities, comparable to Rep2, with ITR2 (60.01%) and increased encapsulation up to 35.43% for ITR6-contained vectors (Figure 6). Our data suggests that Rep engineering can be used as a strategy to improve the production of functional particles.

## 4. Discussion

The choice of AAV serotype as a tool for gene delivery or gene editing depends on the tropism for desired target tissues as well as the ability to generate sufficiently high-quality titers. AAV6-based vectors became commonly used for targeting lymphoid cells [48,49,50], myeloid cells [42,50,51,52], hematopoietic stem cells [53,54], muscular cells [55,56,57], cardiomyocytes [58,59,60], pulmonary cells [44,61] and others.

According to previous studies, ITR3-Rep3 has been used to package AAV3 vectors to produce higher titers than standard ITR2-Rep2 systems, suggesting that matching AAV vectors with wild-type ITRs and the Rep gene could generate better titers and purities than standard protocols [39]. In the current study, we applied a similar strategy to improve packaging on AAV6 vectors. All AAV serotypes had similar Rep78 sequences (85–90%) [38], except for AAV5 Rep78 (58% identity). AAV6 Rep78 protein showed 87% identity when compared with the AAV2 Rep78 protein. Moreover, differences between AAV2 and AAV6 Rep proteins were located in the variable region for Rep proteins of all AAV serotypes, while differences in the binding domain of Rep78/68 were minimal, suggesting the conservative nature of that region. The Rep binding region contains the two pairs of His (H89 and H91), which were conserved and essential for HUH endonuclease activity. The His pair domain of HUH motif provides the coordination site for divalent metal ions (Mg^2+^ or Mn^2+^), which is essential for ssDNA cleavage [62,63,64] and mutations in this region or close to this region could affect DNA recognition and cleavage.

In this study, AAV6 vectors were purified without specific efforts to separate empty and full capsids to understand how non-AAV2 Rep proteins can affect AAV production and packaging. Although each of the wild-type AAV have their own Rep genes, Rep6 did not produce a sufficient amount of AAV6 vectors compared with the commonly used Rep2. This result is consistent with other recent studies that showed low efficiency of Rep6 in production of AAV vectors [65]. Our data suggests that insufficient packaging can be partially explained by lesser Rep78/68 proteins being expressed by the Rep6 gene compared with the Rep2 gene (Figure 4A).

Rep genes are involved in viral genome replication (Rep78/68) and encapsulation into pre-formed capsids (Rep52/40) [20,24,25]. We showed that Rep6 produced AAV6 vectors with approximately a 2-log lower titer and poor full/empty ratios compared with Rep2-AAV6 vectors (Figure 4C and Figure 6). These results correlate with the lack of sufficient expression of Rep78/68, which might lead to insufficient genome replication—which is required for gene encapsulation into AAV capsids.

Next, we showed that mutations on critical amino acids on the N-terminus region of Rep6 (to corresponding amino acids on Rep2) rescued viral productivity per packaging cell, and total viral yield correlated with restored Rep78/68 expression (Figure 4). Rep6-V65T/L90M/I92V mutations increased viral productivity but showed poor full/empty ratios similar to Rep6. Substitution of the four amino acids on Rep6 (V65T; Q66E; L90M; I92V), which we termed as hybrid Rep2/6, restored efficiency of encapsulation to ratios similar to those of Rep2.

Unexpectedly, combining ITR6 and Rep6 to encapsulate AAV genomes into AAV6 capsids that resembled wild-type AAV6 (and presumably provide natural Rep6 activity and p5, p19, and p40 promoter activity) resulted in impaired vector production (Figure 4). When expression in cell lysates from packaging cells were compared at the harvest point, the Rep6/Cap6 and Rep2/Cap6 produced similar amounts of VP proteins (Figure 4A). However, yields from both ITR2- and ITR6-flanked vectors were 50 to 100 times higher than Rep6 (Figure 4C). Additionally, the full/empty ratio was significantly higher in vectors produced with hybrid Rep2/6 compared with Rep6 (Figure 6). These results suggest that VP protein expression and capsid assembly might not be a reason for poor vector yield and quality. Rep78/68 expression was reduced in Rep6 and could be responsible for lower genome replication and sequentially lower genome encapsulation. Our data partially contradicts the results of recent studies that showed low p40 promoter activity in Rep and, thus, lower VP production [38].

Regardless of the high similarity of sequences, Rep2 produced less than 10% of fully encapsulated vectors with the ITR6 flanked expression cassette. The Rep2/6 hybrid, which contained the N-terminus part of Rep2, increases encapsulation of ITR6 vectors compared to Rep6 and Rep 2, although it showed lower levels of encapsulation of ITR2 compared to Rep2 (Figure 6).

AAV2 Rep can be used to package AAV vectors, however, the titer and full/empty ratio depends on the different Rep genes and no rational design and control of expression can fully predict the outcome of Rep activity [38,66,67]. We found that a Rep2/6 hybrid can produce AAV6 vectors with the same in vitro and in vivo transduction efficiency and capsid integrity as standard Rep2, suggesting that the replacement of Rep is only affecting packaging capability and genome encapsulation. Standard AAV6 vectors typically show a 50/50 empty/full ratio, and efforts are focused on optimizing the downstream process to improve the full capsid ratio [68,69]. However, our results highlight that Rep optimization in upstream packaging processes could be another complementary tool to obtain high-titer and high-quality AAV vectors.

## Data Availability

No new data were created or analyzed in this study. Data sharing is not applicable to this article.
